# Protein composition and nutritional aspects of pea protein fractions obtained by a modified isoelectric precipitation method using fermentation

**DOI:** 10.3389/fnut.2023.1284413

**Published:** 2023-11-02

**Authors:** Mehrsa Emkani, Sylvie Moundanga, Bonastre Oliete, Rémi Saurel

**Affiliations:** Univ. Bourgogne Franche-Comté, L'Institut Agro Dijon, PAM UMR A 02.102, F-21000 Dijon, France

**Keywords:** pea albumin, lactic acid bacteria, isoelectric precipitation, peptides, antinutritional factors

## Abstract

Pea albumins are promising for their nutritional, biological, and techno-functional properties. However, this fraction is usually discarded in the industry due to its low protein content compared to globulin fraction and the presence of some anti-nutritional compounds. In the present study, we used an alternative method of pea protein extraction based on alkaline solubilization/isoelectric precipitation in which the reduction of pH was achieved by lactic acid fermentation using specific starters instead of mineral acids. Hence, the main objective of this study was to examine the protein profile and the content of anti-nutritional and nutritional active compounds in pea albumin-rich fractions obtained by the isoelectric extraction method without (control) or with fermentation with different lactic acid bacteria (*Streptococcus thermophilus, Lactiplantibacillus plantarum*, and their co-culture). Different pea cultivars (Cartouche, Ascension, and Assas) were used here for their differences in protein profile. The results revealed a higher total nitrogen content in albumin-rich fraction for fermented samples and, in particular, for co-culture. The majority of total nitrogen was determined as non-protein (~50%), suggesting the degradation of proteins by LAB to small peptides and amino acids, which were solubilized in the soluble fraction (albumin) as confirmed by size exclusion chromatography (SEC-HPLC) analysis. Moreover, the higher antioxidant activity of fermented albumin samples was attributed to the production of small peptides during extraction. Lactic acid fermentation also resulted in a significant reduction of trypsin inhibitor activity, α-galactoside, and phytic acid content of this fraction compared to control.

## 1. Introduction

Over the past decade, there has been a significant interest in pea ingredients for their high protein content, good quality amino acids, and low allergenicity. However, the application of pea protein in the food industry is still challenging because of its low solubility, imperfect sensorial properties, and the presence of antinutritional factors (1–3).

Peas can be used either as grain components (e.g., flour milled from grains) or as enriched protein ingredients such as protein concentrate (50–55% protein) and protein isolate (80–90% protein) (4, 5). The majority of pea proteins are globulins (60–70%) and albumins (15–20%). Due to their unique solubility characteristics, albumin and globulin fractions can be separated and purified. Globulins are salt-soluble, while albumins are water-soluble. Other compounds such as trypsin inhibitors, lipoxygenase, phytate, lectine, and α-galactosides are also soluble in water and are mainly recovered in the soluble albumin fraction after separation from globulin (6, 7). These compounds are considered non-nutritive compounds since they interfere with nutrient availabilities or cause host digestive discomfort or health problems. For instance, trypsin inhibitors are low molecular weight (Mw) proteins capable of binding to the digestive enzyme (i.e., trypsin) and inactivating it, reducing the digestibility of protein, reducing the absorption of amino acids, and reducing the availability of minerals (8). The α-galactosides of sucrose, also known as the raffinose family of oligosaccharides (RFOs), are the second most abundant soluble carbohydrate in legumes (9). α-galactosides are responsible for digestive discomfort and flatulence due to their fermentation by gut bacteria in the large intestine (10). Phytic acid [myo-inositol hexaphosphoric acid (IP6)] consists of a cyclic ring (C_6_H_6_O_6_) where each oxygen is connected to a phosphate group (P(OH)_3_) (11). The unique structure of phytic acid enables it to bind with various molecules such as proteins through different types of interactions, affecting protein solubility (12). Phytic acid can also bind to enzymes and certain minerals, reducing their absorption in the gut. This can lead to iron deficiency anemia in individuals with high daily pulse consumption (13, 14).

Differences in the composition and structure of pea proteins can be achieved due to agronomical conditions (cultivar, weather conditions, etc.) or due to technological processes during extraction or functionalization (physical, chemical, or biological processes). First and foremost, the genetic and phenotypic variation of pea cultivars can affect the ratio of globulins (i.e., 11S/7S) and globulin to albumin fraction (15). The variation of protein fractions can modify the properties of the protein. For instance, the less compact structure of vicilin offers better functional properties compared to legumin (16). Nevertheless, the limited presence of sulfur amino acids in vicilin diminishes its nutritional value (17).

Protein composition and structure are also determined by the extraction method where normally physical and chemical processes are applied. Different methods have been proposed for the extraction of protein from pea flour, including alkaline extraction/isoelectric precipitation (AEIEP), salt extraction dialysis, micellar precipitation, and aqueous extraction (pH > 7) (18, 19). AEIEP is a common technique with a high yield for producing pea protein isolates in the food industry (20). The method separates albumins and globulins since both legumin and vicilin have high solubility at alkaline pH and minimal solubility at their isoelectric point (pI), while albumin remains soluble in a large pH range (21). Globulin fraction obtained by this method is exploited in the form of isolates by industry. Other fractions such as albumins are discarded as by-products.

Biological processes such as lactic acid fermentation can be as important as the other factors (cultivar and extraction methods) in modifying the composition and structure of protein. Indeed, lactic acid bacteria (LAB) during fermentation can have different enzymatic activities such as proteolysis, resulting in the production of small peptides and amino acids (22, 23). The small peptides released by LAB might possess many beneficial health activities such as angiotensin l-converting enzyme (ACE)-inhibitory activity and opioid, antioxidant, antidiabetic, immunomodulatory, and antimicrobial activities (24–26). As a result, fermentation is commonly employed with legume protein ingredients to enhance their physicochemical, nutritional, functional, and sensory properties (26–34).

A recent study in our group has proposed an alternative extraction method based on AEIEP (35). In this method, lactic acid fermentation by using LAB commercial starters was applied during the acid precipitation step. This way, the reduction of pH was obtained thanks to the production of organic acid by specific starters during fermentation, which leads to the precipitation of non-soluble fractions (globulins) and their separation from the soluble ones (albumins). This study showed that the albumin fraction obtained during fermentation had higher protein content than the one obtained by the traditional AEIEP method. Albumin fraction is generally discarded in the industry due to the presence of non-protein soluble compounds and antinutritional components, the possible allergenic activity of this fraction, and, most importantly, the lower content of protein compared to globulin fraction (36–39). This fraction can be interesting from a nutritional point of view since it is considered to have a higher content of sulfur-containing amino acids (40, 41). Moreover, previous studies on pea albumin fraction reported good emulsion and foam-stabilizing properties (42). Especially, interface and foam-stabilizing properties of this fraction seem to be promising compared to globulin, owing to the smaller Mw, lower protein charge, and the specific distribution in hydrophobicity of albumin fraction (39, 43).

Hence, the objective of this study was to evaluate more precisely the effects of fermentation with specific LAB strains on the under-valorized pea albumin fraction obtained with the previously proposed extraction method assisted by fermentation. Protein and peptide contents and other nutritional aspects (α-galactoside and phytic acid contents, and trypsin inhibitor and antioxidant activities) were further discussed in this study. Here, we aimed to use different pea cultivars with contrasted initial protein composition in 7S, 11S, and 2S fractions to observe whether the method could preferentially lead to the enrichment of certain protein fractions. Two LAB strains (*S. thermophilus* and *L. plantarum*) were applied either alone or in co-culture for their promising impact on legume protein properties. *L. plantarum* is a facultative heterofermentative bacteria (44). It is famous for its versatility and its adaptability in different substrates and conditions (45). It has been used numerously in pea ingredient fermentation (27, 28, 46–48). *S. thermophilus* is a homofermentative aerotolerant and is widely used in the dairy industry for its high acidification rate and its contribution to organoleptic properties (49, 50). There are several pieces of evidences of high growth and acidification of this strain in pea substrate, either alone or in co-culture (35, 51–53).

## 2. Materials and methods

### 2.1. Materials

All the chemicals were of analytical grade and supplied by Honeywell FlukaTM (Gillman, SA, Australia) or Thermo Fisher Scientific (Dardilly, France) unless the contrary was indicated. Three different pea cultivars (Cartouche, Ascension, and Assas) with different grain characteristics and polypeptide profiles were used. The cultivars were provided by INRAE (UMR Agroécologie, Dijon, France) as part of the Peavalue project ANR-19-CE21-0008-03. The cultivars were chosen for their protein composition determined by INRAE AgroEcologie laboratory (Table 1). Cartouche (CAR) was rich in vicilins, Ascension (AC) was rich in convicilin, and Assas (AS) was rich in legumin and albumin PA2. Two freeze-dried lactic acid bacteria including, *S. thermophilus* (102303T) (ST) and *L. plantarum* (CNRZ211) (LP), were purchased from the International Center for Microbial Resources-food Associated Bacteria (CIRM-BIA, Rennes, France).

**Table 1:** Dry matter, ashes, and protein contents of pea flour from three cultivars (Cartouche, Ascension, and Assas).

**Genotype**	**Dry matter**	**Ashes**	**Protein content (% db)**	**Polypeptides profile** ^**a**^					
				**11S**	**7S**	**7S/11S**	**PA2**	**Vic/ Conv**	**PA2/ globulins**
Cartouche	90.4 ± 0.0 a^b^	12.5 ± 0.4 a	19.3 ± 0.6 a	21.5	57.2	2.6	15.1	4.0	0.1
Ascension	91.9 ± 0.0 c	12.5 ± 0.4 a	23.6 ± 0.5 b	33.1	50.7	1.5	13.2	2.3	0.1
Assas	91.4 ± 0.0 b	13.0 ± 0.4 a	23.9 ± 0.8 b	42.3	29.0	0.6	23.4	5.2	0.3

11S, legumin; 7S, vicilin/convicilin; PA2, main pea albumin; Conv, convicilin.

a Cultivar characteristics obtained by one-dimensional SDS gel electrophoresis. Data are collected by INRAE (UMR Agroécologie, Dijon, France).

b cultivars flour dry matter, ashes, and protein contents represented as average ± standard deviation, n (n = 3). Different letters in the same row represent significant differences among different samples (Tukey's *post-hoc* test, P < 0.05).

### 2.2. Methods

#### 2.2.1. Bacterial culture

LAB were received freeze-dried. After rehydration in their optimal liquid growth medium (MRS for LP and M17 for ST), the bacteria were incubated for 24 h at the optimal growth temperature of 37°C for LP and 43°C for ST. Cells were then isolated on an agar medium. A single colony was added to 10 mL of liquid medium and incubated for 24 h. A culture was prepared by inoculating 10 mL of broth medium with 1 mL of this pre-culture and was stopped at the beginning of the stationary phase (around 12 h for LP and 7 h for ST). The cell suspension was centrifuged (Eppendorf^®^ Centrifuge 5804/5804R, USA) (4,000 ×g, 20°C, 5 min) and the pellet was resuspended in 1 mL of fresh optimal broth medium. Glycerol 30% was added to this suspension (1:1 ratio), and the contents were transferred to a cryotube. Cryotubes were stored at −80°C as stock culture.

For protein extraction experiments, the contents of a cryotube were transferred to a sterile Eppendorf tube. Glycerol was removed from the bacteria by centrifugation (4,000 ×g, 5 min, 4°C) and replaced by 1 mL of fresh broth medium. The pellet was washed three times. Then, the entire contents were added to 10 mL of fresh broth medium and incubated for 24 h at the optimal growth temperature of the bacteria. After that, 1 mL of pre-culture was added to 10 mL of fresh medium and incubated until the beginning of the stationary phase. Bacteria were harvested at a cell density of 10^7^ CFU/mL for monoculture. For co-culture (STLP), ST and LP were added at a concentration of 0.5 × 10^7^ CFU/mL each in a 1:1 ratio. Bacteria were centrifuged (4,000 ×g, 10 min, 4°C), and the pellet was resuspended in 5 mL phosphate-buffered saline (PBS) before being added to the protein solution.

#### 2.2.2. Pea flour preparation

The seeds from CAR, AC, and AS cultivars were cracked in a Rotor Beater Mill SK300 (RETSCH GmbH, Haan, Germany) to coarsely break grains and separate the hull. The hull was removed by blowing air. Dehulled cracked grains were milled in the same Rotor Beater Mill SK300 (RETSCH GmbH, Haan, Germany) up to a particle size of <1 mm. Flours from the different varieties were then sieved (<800 μm). Table 1 represents the characteristics of pea cultivars flour. The protein content of flour was determined by the Kjeldahl method [conversion factor 5.4 (54)]. Ashes and dry matter contents of flour were determined by the 942.05 AOAC method (55) and the 935.29 AOAC method (56), respectively.

#### 2.2.3. Pea protein extraction

**Figure 1:**
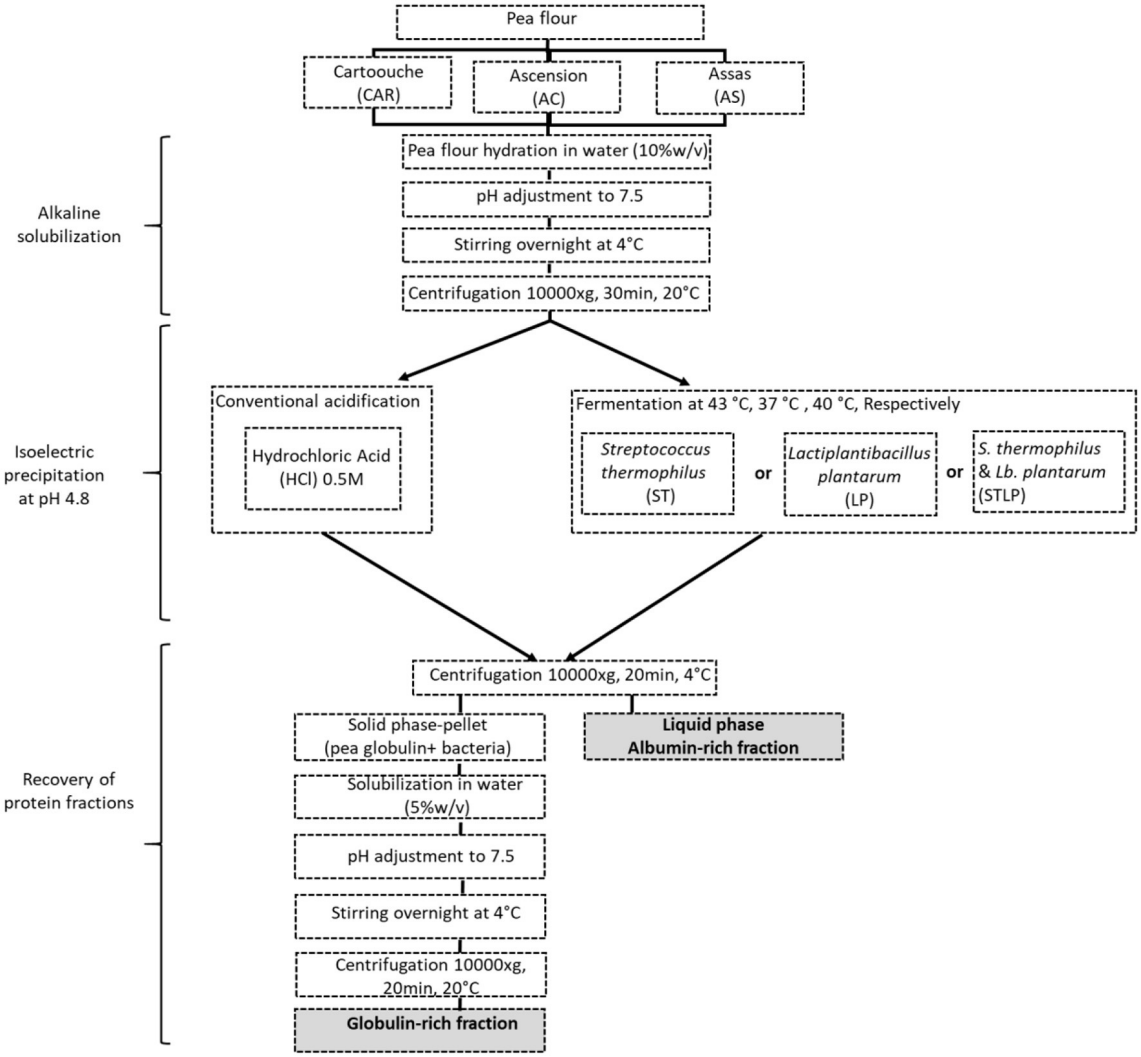
Schematic view of pea protein extraction.

The method of extraction is shown in Figure 1.

The extraction method followed the protocol proposed by Emkani et al. (35). In brief, the pea flour was mixed with water (10% w/w), and the pH was adjusted to 7.5 by NaOH (0.5 M) addition. The solution was stirred overnight, and the pH was readjusted to 7.5. Insoluble material was removed by centrifugation (10,000 ×g, 30 min, 20°C), and the supernatant (protein extract) was collected. Acidification up to the isoelectric point of globulins (pH=4.8) was applied to separate the globulin fraction from the albumin fraction. The conventional acidification method used HCl addition. In the newly proposed method, acidification was achieved by fermentation. The selected lactic acid bacteria were added to the protein solution either as mono- or co-culture. While the pH was monitored automatically, the protein solution was incubated at an optimal growth temperature of the bacteria, which was 37, 43, and 40°C for LP, ST, and STLP, respectively. The acidification was stopped at pH 4.8, which corresponds to the isoelectric point of the globulins (57). The soluble part which is the albumin fraction was separated from the non-soluble part by centrifugation (10,000 ×g, 30 min, 4°C). The obtained pellet contained mainly globulins together with biomass. To obtain the globulin-rich fraction, the pellet was solubilized in water (5% w/v), and the pH was adjusted to 7.5 by NaOH 0.5 M. The sample was stirred overnight and the pH was readjusted to 7.5. The solution was again centrifuged (10,000 ×g, 20 min, 20°C), and the supernatant was collected. All the samples were freeze-dried (Heto PowerDry PL6000, Thermo Scientific, Waltham, MA, USA) and stored at −5°C until analysis. The freeze-drying conditions were the same as indicated by Oliete et al. (58). The total nitrogen and non-protein nitrogen content of the obtained protein fractions was determined by the Kjeldahl method according to Emkani et al. (35).

#### 2.2.4. Acidification kinetic parameters

The acidification kinetic parameters were characterized according to Spinnler and Corrieu (59). The evolution of pH in protein extract was measured automatically at 5 min intervals (pH meter 3310, WTW GmbH, Weilheim, Germany) and carried out in triplicates. The time variation of pH (dpH/dt) was then calculated, and the maximum rate of acidification (V_max_) was expressed as pH units/h. The other kinetic parameters include time (t_vmax_) and pH (pH_Vmax_) at which the maximum acidification rate was observed, final pH (pH_f_) at which the pH was stable, and time (t_pHf_) and (t_pH4.8_) required to reach pH_f_ and pH 4.8, respectively, were also measured.

#### 2.2.5. SDS polyacrylamide gel electrophoresis

The polypeptide composition of albumin and globulin fractions was characterized by SDS-PAGE for all the extraction conditions. NovexTM electrophoresis gels at 10%−20% Tris-Glycine were used. Samples were diluted by at least half in the sample buffer: 187.5 mM Tris-HCl, pH 8.9, 10% (w/v) glycerol, 2% (w/v) SDS, and 0.05% (w/v) bromophenol blue, in the presence (reducing conditions) or absence (non-reducing conditions) of 2% (w/v) dithiothreitol (DTT). The samples under reducing conditions were heated in a water bath for 10 min at 95°C. All the samples were prepared and then deposited in the wells of the gel to have 10 μg of protein per well. The Mw protein markers from Sigma–AldrichR (SigmaMarkerTM S8445, wide range, Mw 6.5 to 200 kDa) were used for all samples except the samples obtained by STLP, which were obtained from Thermo Fisher Scientific (Thermo Scientific™, PageRuler™ unstained broad range protein ladder, Mw 5 to 250). The migration was carried out at 35 mA per gel, with the following migration buffer: 0.3% (w/v) trizma base, 1.45% (w/v) glycine, and 0.1% (w/v) SDS, in a ScientificR Mini Gel Tank of Migration (Thermo Fisher Scientific Inc.). The gels were then rinsed with distilled water, and the fixation was performed in four successive distilled water baths heated for 1 min in a microwave at 550 W. The staining of the gels was performed with Coomassie blue, Thermo Scientific™ PageBlue™ Protein Staining Solution, overnight. The discoloring was then achieved in several baths of distilled water, until the desired color. The gels were then scanned using the Odyssey infrared imaging system (LI-COR Biosciences, https://www.licor.com). Protein band detection was performed using CLIQS (TotalLab, http://www.totallab.com).

#### 2.2.6. Size exclusion chromatography (SEC-HPLC)

The size distribution of the peptides in the albumin fraction was determined by high-pressure liquid chromatography (HPLC Shimadzu Corporation, Kyoto, Japan). The HPLC system was equipped with an isocratic pump (Shimadzu LC-20AT), a UV-visible detector (Shimadzu SPD-20AV), and a size exclusion column, as Protein-Pak SEC Column, 60 Å, 10 μm (7.8 mm X 300 mm, 500–20K) (Waters, Milford, MA). The column was equilibrated at 25°C with a mobile phase, including a phosphate buffer (Na_2_HPO_4_) 100 mM and a pH 7 containing 0.3 M NaCl filtered through a 0.22-μm Durapore^®^ Membrane Filter (filter hydrophilic PVDF, 47 mm membrane) (Sigma Alrich, Merk SA, Darmstadt, Germany). The column was pre-calibrated by the protein standards including Insulin B chain, Leucine, Myoglobin, and Cytochrome C from a bovine heart, supplied by Sigma Aldrich (Merk SA, Darmstadt, Germany) to determine the elution volume. Solution of standard and freeze-dried protein samples were dissolved in filtered phosphate buffer (Na_2_HPO_4_) 100 mM and a pH 7 containing 0.3 M NaCl with a concentration of ~1 mg/mL. The Mw fraction less than 10 kDa was separated from the protein solution by centrifugal filters (Amicon Ultra-15 Centrifugal Filter Unit, Merk SA, Darmstadt, Germany), and then they were filtered through a syringe filter (0.45 μm, 13 mm, Restek France, Lisses, France). The standard and protein solutions were then injected (20 μl) at a flow rate of 0.1 mL.min^−1^ for 120 min. The absorbance was then measured at different wavelengths 214, 280, and 254 nm. The wavelength at 254 was used to assess the interference with phenolic compounds. The best results were achieved at 214 nm due to the better absorption coefficient of protein. Tests were performed in triplicate. A calibration curve was obtained over a range of 0.3 to 16.7 kDa. The calibration curve equation and the correlation coefficients were y = −0.18x + 1.503, R^2^ = 0.986. Chromatograms were recorded and processed by LabSolution LC (HPLC Shimadzu Corporation, Kyoto, Japan).

#### 2.2.7. Sugar quantification

Oligosaccharides of the raffinose family (RFOs), consisting of raffinose, stachyose, and verbascose, as well as the sucrose and D-glucose contents, were measured in the protein solution after alkaline solubilization and in the albumin fractions by an enzyme-based assay kit (Megazyme Raffinose/d-Glucose Assay Kit, Megazyme International, Ireland). The kit consisted of α-galactosidase (from *A. niger*), invertase (from yeast), and glucose determination reagent, i.e., glucose oxidase/peroxidase (GOPOD) for colorimetric estimation of sucrose and RFOs contents. The kit is based upon the principle of stepwise hydrolysis of complex soluble carbohydrates to glucose followed by its colorimetric measurement. Soluble sugars such as sucrose and RFOs were hydrolyzed with α-galactosidase and invertase into D-glucose, D-galactose, and D-fructose. D-glucose concentration was determined using GOPOD reagent. The concentration of raffinose, stachyose, verbascose, and other higher homologs of the RFOs in samples was measured as a group because α-galactosidase hydrolyses all members of the RFO family. Since 1 mole of each of the RFO contains 1 mole of D-glucose, the RFO concentrations were presented on a molar basis (mmol/100 g sample).

In brief, 0.5 g of each sample was treated with 95% ethanol (to digest the endogenous enzymes completely) at 85°C for 20 min, and the final volume was made up to 50 mL using sodium acetate buffer (50 mM, pH 4.5). The obtained digested mixture was incubated at room temperature for 20 min and vortexed to obtain a uniform slurry. Subsequently, 2 mL chloroform was added to the 5-mL slurry obtained and vortexed for 15 s followed by centrifugation at 1,000 ×g for 10 min. A volume of 0.2 mL from the aqueous phase of the supernatant was taken in three tubes (namely, A, B, and C). A volume of 0.2 mL sodium acetate buffer (50 mM, pH 4.5), 0.2 mL of invertase (8.3 U/mL), and a mixture of invertase + α-galactosidase (invertase 8 U/mL and α-Galactosidase 40 U/mL) was added into tubes A, B, and C, respectively. All three tubes were incubated at 50°C for 20 min. Reagent blank (0.4 mL sodium acetate buffer) and glucose control (0.1 mL standard glucose solution, which contained 0.556 μmol of glucose + 0.3 mL sodium acetate buffer) were also taken simultaneously. Subsequently, 3 mL of GOPOD reagent was added in all of the tubes and incubated again at 50°C for 20 min. The glucose concentration for tubes A, B, and C and glucose control was determined by measuring the change in absorbance at 510 nm against the reagent blank using a spectrophotometer (UV/Visible Jenway 6305, Barloworld Scientific, Dunmov, UK). Glucose, sucrose, and RFOs concentrations were shown in mmol/100 g sample. The concentrations of glucose, sucrose, and RFOs were calculated as follows:


(1) Glucose (mmol/100 g) = ΔA × F × 250 × 200 × 1/1000

(2) $$\begin{array}{l} Sucrose\;(mmol/100\;g)\\ =\;(\Delta B-\Delta A)\;\times \;F\;\times \;250\;\times \;200\;\times \;1/1000 \end{array}$$

(3) $$\begin{array}{l} RFOs\;\left(mmol/100g\right)\\ =\left(\Delta C-\Delta B\right)\times \;F\;\times \;50\;\times \;250\;\times \;200\times 1/1000, \end{array}$$

where ΔA, ΔB, and ΔC were the absorbance of the sample plus sodium acetate buffer, sample plus invertase, and α-Galactosidase enzyme solution, respectively.

F = Factor to convert from absorbance to μmol of glucose= 0.556 (μmol of glucose)/GOPOD absorbance for 0.556 μmol of glucose; 250 = conversion to 50 mL of extract; 200 =conversion from 0.5 to 100 g of sample; and 1/1,000 = conversion from μmol to mmol.

All enzymatic assays were performed in three technical replicates (n = 3) for each sample. The consumption patterns of D-glucose, sucrose, and RFOs in the albumin fraction of samples obtained without fermentation (control) and with added fermentation by ST, LP, and STLP were shown as a ratio calculated from the content of these sugars in the initial protein extract.

#### 2.2.8. Free amino groups

The content of the free amino group was measured by trinitrobenzene sulfonic acid (TNBS) following the method of Adler-Nissen (60). In brief, globulin and albumin fractions with a concentration of 50 mg protein/mL (measured by the Kjeldhal method with a nitrogen conversion factor of 5.4) were prepared in phosphate buffer 0.1 M pH 8.2, 2% SDS w/v. A volume of 250 μL of the sample was added to 2 mL of TNBS reagent (0.5 g/L) and 1.75 mL phosphate buffer 0.2 M pH 8. The TNBS reagent was prepared immediately before use. This solution was incubated for 60 min at 50°C, and the absorbance at 340 nm was measured after the addition of 4 mL HCl 0.1 M to stop the reaction. A standard curve was obtained by sing L-leucine (at a concentration of 0–3 mM) as control.

#### 2.2.9. Determination of antioxidant activity

Trolox equivalent antioxidant capacity (TEAC) was analyzed in albumin fractions by a total antioxidant capacity assay kit (MAK187, Sigma-Aldrich, Merk SA, Darmstadt, Germany) according to the manufacturer's instructions. The method is based on the reduction of Cu^2+^ to Cu^+^ by antioxidant molecules, which give an absorbance at 570 nm. In brief, 100 μL of albumin fractions were added to a 96-well plate with a clear flat bottom. Different dilutions of samples were also prepared and the volume was adjusted to 100 μL by distilled water to ensure that the readings were within the standard value range. Then, 100 μL of Cu^2+^ reagent was added to the samples and incubated in darkness at room temperature for 90 min, and the absorbance was measured at 570 nm using a microplate reader (Paradigm Detection Platform, Beckman Coulter, Harbor, Oregon, USA). Trolox solutions ranging from 0 to 20 nmol per well were used to prepare a calibration curve. The antioxidant activity was expressed as nmol Trolox equivalents per μL sample (nmol Trolox/μL).

2,2-diphenyl-picrylhydrazyl (DPPH) free radical scavenging ability was performed according to the method proposed by Brand-Williams et al. (61). In this assay, antioxidant compounds present in the sample reduced the DPPH· radicals, which had an absorption maximum of 517 nm. The DPPH· radical solution was prepared by dissolving 10 mg of DPPH in 25 mL of 80% methanol. First, the extinction of the disposable cuvette with 250 μL of the methanolic DPPH· solution and 2.1 mL of 80% methanol was measured as blank. Then, 100 μL of the sample was added to 250 μL of the methanolic DPPH· solution and 2 mL of 80% methanol. The mixture was shaken and allowed to stay at room temperature in the dark for 20 min. The decrease in absorbance of the resulting solution was monitored at 517 nm for 20 min using a spectrophotometer (UV/Visible Jenway 6305, Barlo world scientific, Dunmov, UK). The results were expressed as a percentage of reduction of the initial DPPH absorption.

#### 2.2.10. Trypsin inhibitor analysis

Trypsin inhibitory activity of pea flour and albumin fraction was determined following the method described by Smith et al. (62) with some modifications. In brief, 10 mg of finely ground pea flour or freeze-dried albumin fraction was mixed with 5 mL of NaOH 10 mM (pH adjusted to ~9 by NaOH 1M) for 3 h at room temperature. The solution (called extract shown by V) was centrifuged (10,000 ×g, 30 min, 20°C), and the supernatant was separated. Trypsin (20 μg/mL) (trypsin from bovine pancreas, Merck/MilliporeSigma, Burlington, MA, United States) solution was dissolved in Tris-HCL buffer (20 mM pH 7.5). An amount of 200 μL of prepared sample was mixed with 200 μL of trypsin and incubated for 10 min at 37°C. The reaction started by the addition of 500 μL of 1 mM Nα-benzoyl-DL-arginine-ρ-nitroanilide (BApNA) (Merck/MilliporeSigma, Burlington, MA, United States) prepared in 1% (v/v) of dimethyl sulfoxide and Tris-HCL buffer (20 mM pH 7.5). The BApNA reagent was prepared immediately before use. The assay tubes were then incubated for 10 min at 37°C. The reaction was stopped by adding 100 μL of 30% acetic acid (v/v). It was then centrifuged at 2,000 ×g for 10 min. The absorbance of samples was measured at 410 nm and symbolized as. The absorbance was compared to a trypsin standard, which was determined by using the same procedure except for replacing the extracted trypsin inhibitors with water. The corresponding absorbance was symbolized as Ac. A trypsin inhibitor unit (TIU) was defined as an increase of 0.02 absorbance at 410 nm. With this definition, trypsin inhibitor activity (TIA) is defined as TIU per mg sample and it was calculated as follows:


(4) $$\begin{array}{l} \text{TIA}=\text{TIU}/\text{mg sample}=\left\{\frac{\left[\frac{Ac-\frac{As}{Ac}}{0.02}\times V\;\right]}{m}\right\} \end{array}$$

where As was the absorbance of the sample, Ac was the absorbance of standard, V was the volume (mL) of extract (5 mL), and m was the mass (mg) of the sample. Trypsin inhibitor activity assay was performed in triplicate.

#### 2.2.11. Phytic acid content

The determination of the phytate content of pea flour and albumin fractions was done according to the method developed by Davies and Reid (63) with some modifications. In brief, 0.5 g of pea flour and freeze-dried albumin samples were mixed with 20 mL of HNO_3_ 0.5 M, and the suspensions were put under continuous stirring for 3 h. Each sample was then filtered with a Whatman No.1 filter paper to obtain the extract. The stock solution of ferric ammonium sulfate (FAS) (2.16 mg/mL) was prepared freshly. The working solution was prepared by diluting one volume of stock solution into 24 volumes of distilled water. Then, 0.2 mL of the previous extract was mixed with 0.2 mL of the working solution of FAS in a test tube, and the test tube was kept in a boiling water bath for 20 min. After the tube cooled to room temperature, 1 mL isoamyl alcohol was added to the tube followed by 0.02 mL of ammonium thiocyanate (5 g/50 mL). The tube was centrifuged (3,000 ×g, 10 min). Finally, the intensity of the color in the isoamyl alcohol layer was determined at 465 nm using a spectrophotometer against an isoamyl alcohol “blank”, exactly 15 min after the addition of the HN_4_CNS. Since the principle of this method is based on an indirect measurement of phytic acid, the idea is to precipitate the ferric ion complex with phytate at acidic pH. The excess of ferric ions will later make a characteristic pink complex with thiocyanate ions. The extinction at 465 nm in the amyl layer is inversely related to the phytate anion concentration. The phytate content can be obtained by reference to a calibration curve prepared with the same quantities of iron, thiocyanate acid, and a standard phytate preparation. The standard stock solution was prepared by dissolving 50 mg sodium phytate in 20 mL of distilled water and making the final volume of 100 mL with distilled water. The working solution was of 0.5 mg/mL concentration. Under these conditions, an inverse linear relationship was found over a range of 0 to 500 μg of phytate.

#### 2.2.12. Statistical analysis

One-way analysis of variance (ANOVA) was performed using Statistica software, version 12 (Tulsa, OK, USA). Tukey's *post-hoc* least significant differences method was used to describe means with 95% confidence intervals.

## 3. Results and discussion

### 3.1. Acidification kinetics and sugar consumption

**Figure 2:**
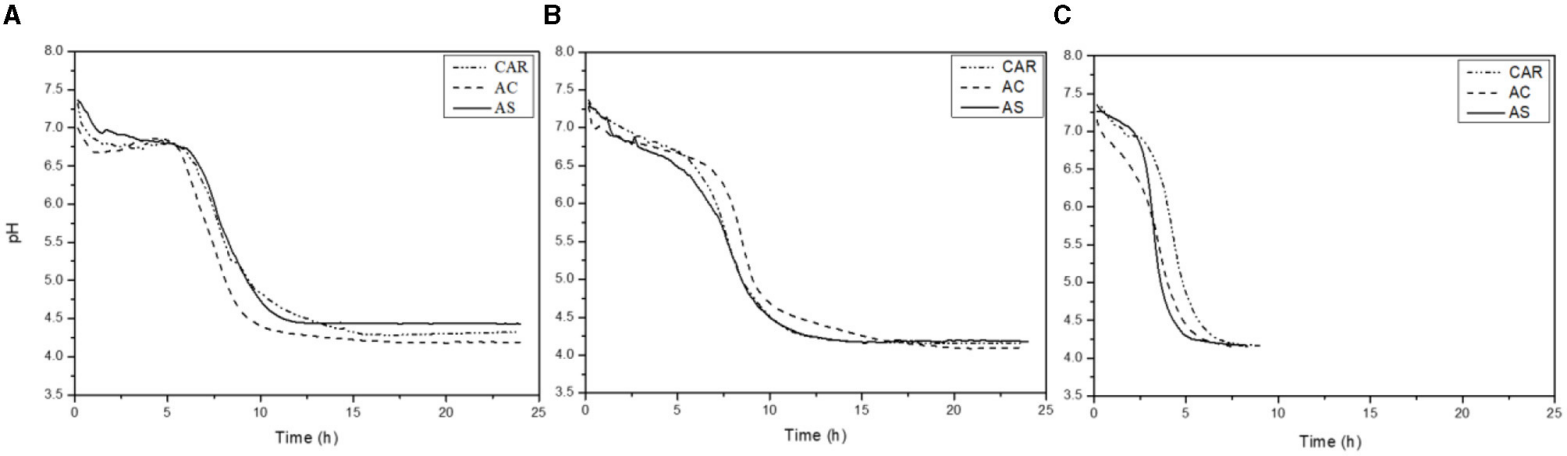
Acidification kinetics of LAB strains [*S. thermophilus* (ST) **(A)**, *L. plantarum* (LP) **(B)**, and *S. thermophilus*+ *L. plantarum* (STLP) **(C)**] in protein extracts obtained from different pea cultivars (CAR, AC, and AS).

Figure 2 shows the pH evolution during the fermentation of soluble pea protein extracts obtained from pea flours of three different varieties (CAR, AC, and AS) by ST, LP, and their co-culture. The diagrams showed that pea protein suspensions were a suitable substrate since all the bacteria were able to grow and reduce the pH. However, the time required to reduce the pH was quite different between the strains.

Table 2 shows the acidification kinetic parameters in protein extracts from three pea cultivars fermented with mono- or co-culture. The highest acidification rate (V_max_~1.2–2.5 pH units/h) and lowest t_Vmax_ (~3.6 h) values were related to the co-culture compared to the mono-cultures (V_max_ ~0.8–0.9 pH units/h; t_Vmax_=7.5–8.5 h) with significant differences. The highest V_max_ value (2.5 pH units/h) in co-culture was related to the variety AS. While there was no significant difference between the varieties in mono-cultures, the pH_Vmax_ represents the pH at the maximum acidification rate, corresponding to the maximum acid-producing ability of the strains. There was no significant difference in pH_Vmax_ (~5–6) and between any variety fermented either with mono- or co-cultures. The pH_f_ represents the final reducing pH after which the pH was stable. The value of pH_f_ was higher with ST (~4.2–4.4) compared to LP and co-culture (~4.1), indicating lower acidification. The time required to reach this pH (t_pHf_) was higher in mono-cultures (~14–16 h) compared to co-culture (~7–7.5 h). The t_pH4.8_, indicating the time required to reach pH 4.8 (isoelectric point of globulins), had a higher value with ST (~9.5–10.1 h), followed by LP (~9–9.5 h). Co-culture (~3.9–4.7 h) showed the lowest t_pH4.8_ compared to the mono-cultures.

**Table 2:** Acidification kinetic parameters for fermented pea protein suspensions obtained from flours of different varieties (CAR, AC, and AS) with mono-cultures and co-cultures of *S. thermophilus* (ST) and *L. plantarum* (LP).

**Strain**	**Pea cultivars**	**V_max_ (pH units/h)**	**t_Vmax_ (h)**	**pH _Vmax_**	**pH f**	**t_pHf_ (h)**	**t_pH 4.8_ (h)**
ST	CAR	0.8 ± 0.0ab	8.1 ± 0.9cd	5.6 ± 0.4a	4.2 ± 0.1b	15.3 ± 0.9bc	10.1 ± 0.1c
	AC	0.8 ± 0.0a	7.5 ± 1.5b	5.7 ± 0.5a	4.2 ± 0.1ab	15.5 ± 0.4bc	9.5 ± 0.3bc
	AS	0.8 ± 0.0a	7.5 ± 1.1b	6.0 ± 0.7a	4.4 ± 0.1c	14.3 ± 0.8b	10.0 ± 0.2c
LP	CAR	0.8 ± 0.1a	8.33 ± 1.4d	5.5 ± 0.3a	4.1 ± 0.1a	14.6 ± 0.4b	9.0 ± 0.2b
	AC	0.9 ± 0.1ab	8.5 ± 1.3d	5.5 ± 0.5a	4.1 ± 0.1a	16.1 ± 1.2c	9.5 ± 0.3bc
	AS	0.8 ± 0.0a	8.1 ± 0.8cd	5.4 ± 0.3a	4.1 ± 0.1a	14.1 ± 0.5b	9.0 ± 0.2b
STLP	CAR	1.5 ± 0.1c	3.75 ± 0.9a	6.1 ± 0.5a	4.1 ± 0.0a	7.5 ± 0.2a	4.7 ± 0.6a
	AC	1.2 ± 0.2bc	3.5 ± 1.0a	5.5 ± 0.2a	4.1 ± 0.1a	7.0 ± 0.2a	4.1 ± 0.7a
	AS	2.5 ± 0.2d	3.6 ± 1.1a	5.9 ± 0.3a	4.1 ± 0.0a	6.8 ± 0.5a	3.9 ± 0.6a

V_max_, maximum rate of acidification; t_Vmax_, the time at which the maximum acidification rate was observed; pH _Vmax_, pH at which the maximum acidification rate was observed; pH f, final pH; t_pHf_, the time required to reach final pH; t_pH 4.8_, the time required to reach pH 4.8.

^a^ The values were presented as average ± standard deviation, n (n = 3). Different letters in the same column represent significant differences among different samples (Tukey's *post-hoc* test, P < 0.05).

The highest V_max_, the lowest t_Vmax_, pH_f_, t_pHf_, and t_pH4.8_ observed in the co-culture could be explained by the synergetic effect of the combined bacteria culture. Probably, the secretion of bioactive substances by one or both LAB used in the study would improve the growth performance and the acid lactic production. This behavior has been pointed out by many authors. Mishra and Mishra (64) observed that both *S. thermophilus* and *L. plantarum*, once in combination, resulted in an increased rate of fermentation and reduced fermentation time. Emkani et al. (35) showed the mixed culture of *S. thermophilus*, with *L. acidophilus* and *B. lactis* having a better acidification profile in pea protein compared to the mono-cultures. Li et al. (65) reported that the combination of *S. thermophilus* and *L. plantarum* in milk fermentation had higher pH reduction compared to the co-culture of *S. thermophilus* and *B. lactis*. The higher value of pH_f_ and t_pH4.8_ in ST could be explained by the lower acidification capacity of this strain compared to LP. Indeed, the acidification capacity is a strain-dependent metabolic feature that could be influenced by many factors such as the metabolism of sugar and the proteolytic system (50, 66). Metabolism of sugar by LAB leads to the production of organic acids and reduction of pH.

**Figure 3:**
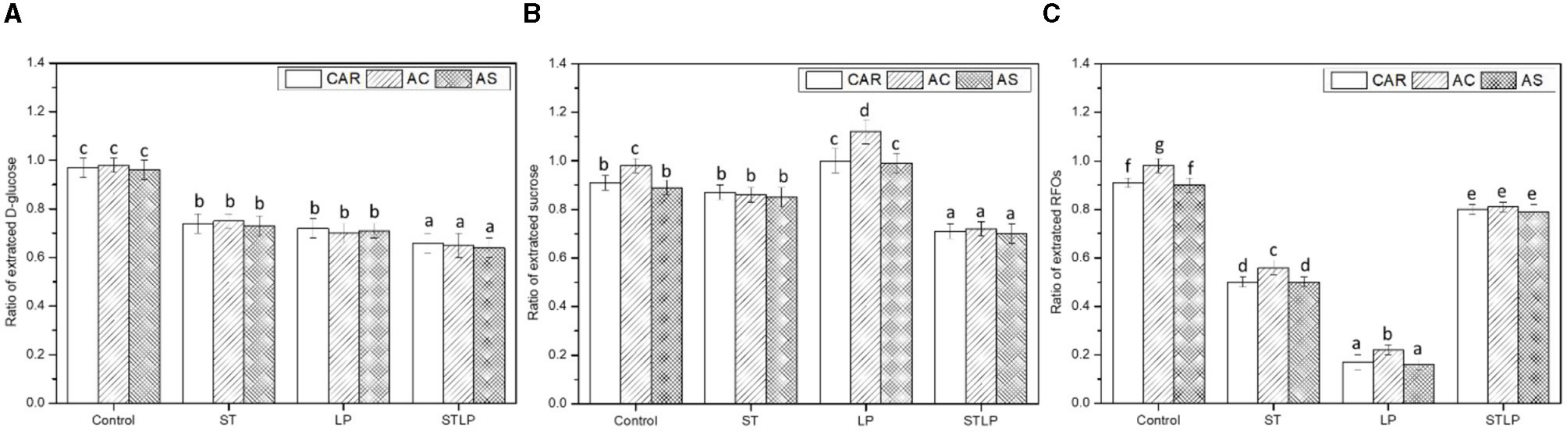
Ratio of recovered glucose **(A)**, sucrose **(B)**, and RFOs **(C)** of albumin fractions obtained without (control) or with added fermentation by *S. thermophilus* (ST), *L. plantarum* (LP) or their mixture (STLP), calculated from initial protein extract. Comparison between different pea cultivars: CAR, AC, and AS. Different letters represent significant differences among different samples (Tukey's *post-hoc* test, *P* < 0.05).

Therefore, the ratio (relative content) of recovered glucose, sucrose, and RFOs (Figures 3A–C, respectively) was measured in albumin fractions obtained without (control) and with fermentation with ST, LP, and their co-culture (STLP) and was calculated from the initial protein extract of different pea cultivars (CAR, AC, and AS). In control, the ratio of these carbohydrates was close to 1. Indeed, these carbohydrates are all water soluble, and their majority is supposed to be found in albumin fraction after the acidification step. The content of glucose, sucrose, and RFOs in albumin fractions obtained by control was ~0.038 M (6.8 g/L), 0.016–0.02 M (5.4 g/L), and 0.03–0.045 M, respectively. No significant differences were observed in the content of glucose between the cultivars obtained by control. However, cultivar AC had a higher relative content of sucrose and RFOs in control.

In fermented samples, LAB were able to reduce the content of these sugars by consuming them as a source of energy for growth, causing the release of organic acids. The relative content of glucose was reduced by all the strains (Figure 3A). The lowest value of glucose and sucrose was related to STLP. The ability of *S. thermophilus* and *L. plantarum* to consume glucose was reported previously (49, 67). Both ST and LP had α-galactosidase activities since they were able to reduce the content of RFOs (Figure 3C). The α-galactosidase activity of *S. thermophilus* (68, 69) and *L. plantarum* (70, 71) have been shown previously. However, the activity of this enzyme seemed to be strain-dependent. The highest consumption of α-galactosides was associated with the fermentation with LP. The reduction of RFOs was ~70% in LP and ~40% in ST, regardless of the pea cultivar. In samples obtained by ST and LP, cultivar AC had a higher content of RFOs compared to the two other cultivars. The lowest α-galactoside consumption was observed for the co-culture (<20%). In general, the optimum pH for bacterial α-galactosidases is in the range of 6.0–7.5 (72). It could be then suggested that the rapid reduction of pH in co-culture limited the action of this enzyme. The decrease of pH in the co-culture would be thus related to the consumption of the other soluble carbohydrates present in pea flour, which was higher compared to the mono-culture samples (Figures 3A, B). Simultaneously to the α-galactoside content reduction, an increase (~10%) in sucrose content was measured for LP, especially with cultivar AC (Figure 3B). This result could be related to the combination of two effects: (i) the noticeable reduction in RFO content increased sucrose release due to the α-galactosidase activity and (ii) LP is not able to catabolize sucrose efficiently as already indicated by Wang et al. (73). Moreover, no significant change in sucrose content was observed for ST compared to control. It is known that *S. thermophilus* species can consume sucrose (49). The consumption of sucrose by ST would be counteracted by the release of sucrose resulting from the hydrolysis of RFOs. The increase in the content of sucrose during fermentation has been previously reported for the fermentation of soymilk by *L. fermentum* (74).

### 3.2. Total and non-protein nitrogen contents

**Figure 4:**
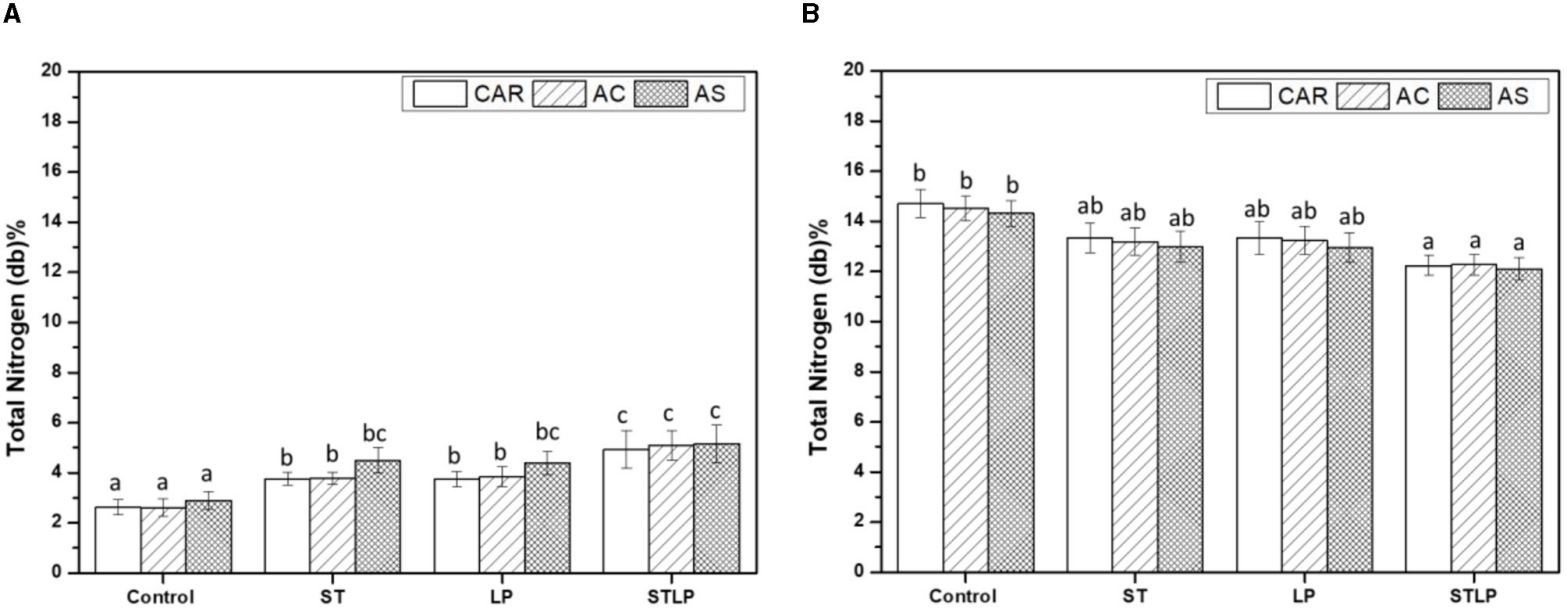
Total nitrogen content of albumin **(A)** and globulin **(B)** fractions obtained without (control) or with added fermentation by *S. thermophilus* (ST), *L. plantarum* (LP), or their mixture (STLP). Comparison between different pea cultivars: CAR, AC, and AS. Different letters represent significant differences in total nitrogen content among different samples (*P* < 0.05).

The isoelectric precipitation extraction method was applied to recover albumin and globulin fractions from pea flour of three different cultivars (CAR, AC, and AS) without (control) or with fermentation using mono-cultures of ST and LP and their co-culture. The total nitrogen (TN) content of albumin and globulin fractions was shown in Figure 4. The results indicated that, in albumin fractions (Figure 4A), TN content was significantly higher for fermented samples (~4–5%) compared to control (~3%). Moreover, co-culture (~5%) had higher values compared to mono-cultures (~3.7–4.5%), while there was no significant difference between the mono-cultures of ST and LP. Comparing pea varieties, it seemed that AS (~4.5%) fermented with both ST and LP had the highest content of TN in albumin fraction. This is not surprising since the variety AS was initially richer in PA2. Regarding the globulin fractions (Figure 4B), the TN content was higher in control (~14.3–14.7%) compared to the fermented samples with co-culture (~12%) having the lowest value. This result was coherent with what was observed in albumin fractions. The decrease in the TN content of globulin fraction was supposed to result from the hydrolysis of some pea proteins to smaller polypeptide chains, which probably solubilized during the extraction process and were recovered in the albumin fraction.

**Figure 5:**
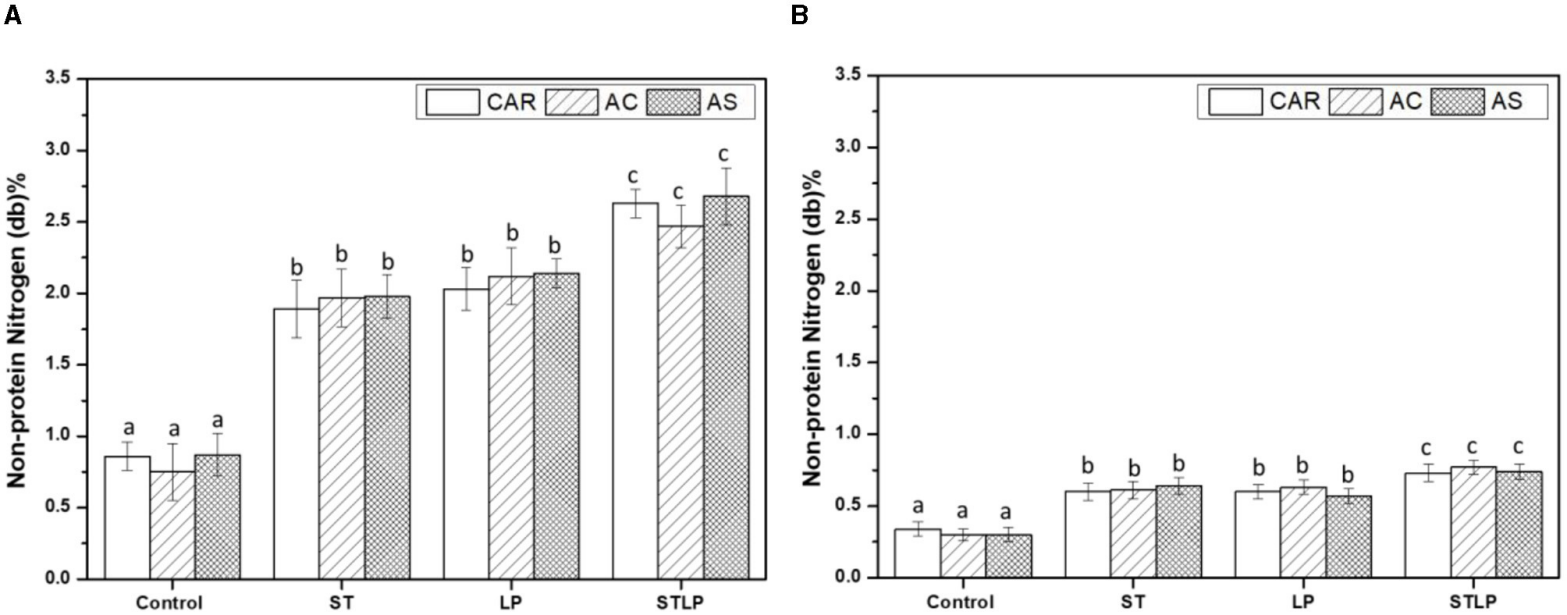
Non-protein nitrogen content of albumin **(A)** and globulin **(B)** fractions obtained without (control) or with added fermentation by *S. thermophilus* (ST), *L. plantarum* (LP), or their mixture (STLP). Comparison between different pea cultivars: CAR, AC, and AS. Different letters represent significant differences in non-protein nitrogen content among different samples (Tukey's *post-hoc* test, *P* < 0.05).

Non-protein nitrogen (NPN) content of albumin (Figure 5A) and globulin (Figure 5B) fractions of pea cultivars obtained by both control and fermentation was measured. The results showed that fermented samples had the highest content of NPN compared to the control (albumin: ~0.8% and globulin: ~0.3%) in both fractions. In addition, the co-culture (albumin: ~2.6% and globulin: ~0.7%) sample had the highest values in both protein fractions compared to the mono-culture ones. NPN in fermented samples represented ~5% and 50% of TN in globulin and albumin fractions, respectively, which was approximately two-fold higher than control. An increase in the content of NPN could be explained by the hydrolysis of protein during fermentation (75–77). Generally, the content of NPN depends on cultivars (78). However, in this study, there were no significant differences between the varieties.

It is worth saying that, despite the application of fermentation in the isoelectric precipitation step, the total protein content in globulin fractions was ~72% in fermented samples compared to ~77% in control, considering the N-to-protein conversion factor of 5.4. By applying an N-to-protein conversion factor of 6.25 classically used for commercial pea protein isolates, these contents were ~83% and 88%, respectively, in the range of values usually observed by applying the AEIEP method (18).

**Figure 6:**
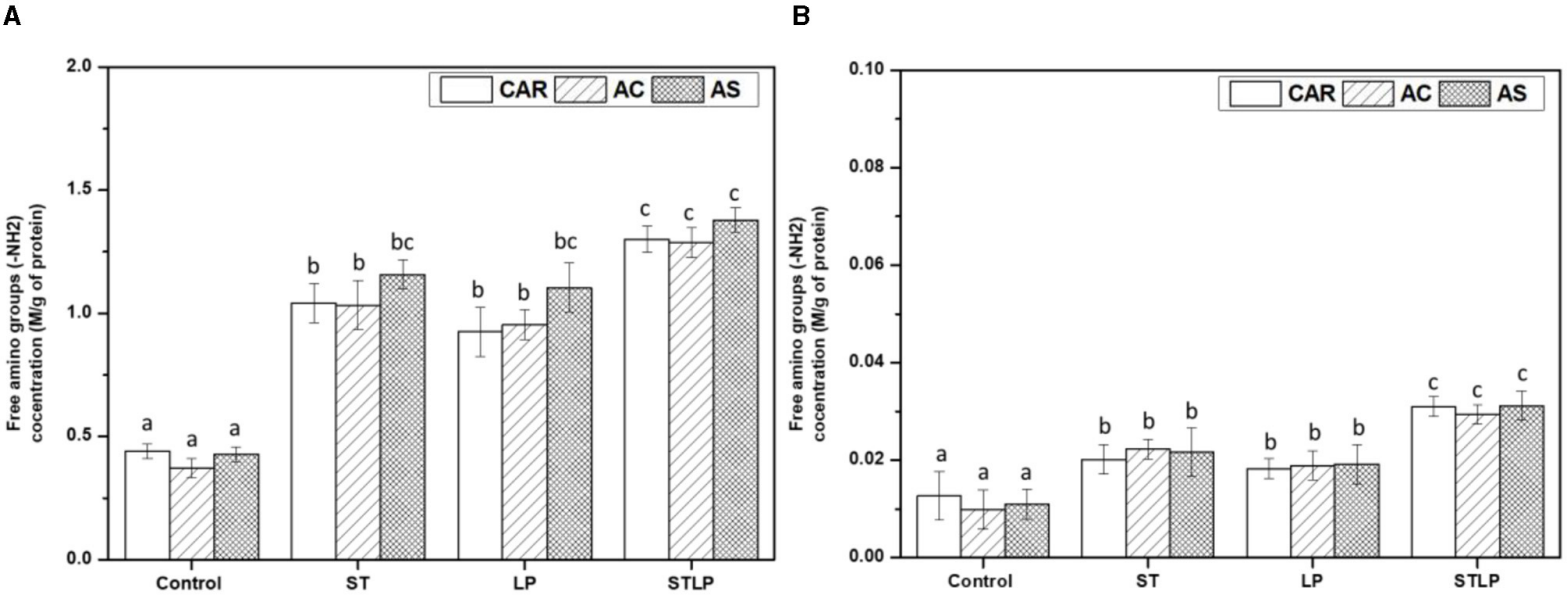
Free amino group content of albumin **(A)** and globulin **(B)** fraction obtained without (control) or with added fermentation by *S. thermophilus* (ST), *L. plantarum* (LP), or their mixture (STLP). Comparison between different pea cultivars: CAR, AC and AS. Different letters represent significant differences in free amino group content among different samples (Tukey's *post-hoc* test, *P* < 0.05).

Free amino groups were measured to evidence the proteolysis effect occurring in fermented samples. The content of free amino groups in albumin and globulin fractions obtained without (control) or with fermentation with ST, LP, and their co-culture from different pea cultivars (CAR, AC, and AS) was shown in Figures 6A, B, respectively.

Free amino group content was significantly lower in control albumin (~0.4 M/g protein) and globulin (~0.01 M/g protein) fractions compared to the fermented samples in agreement with the previous NPN data. Additionally, the samples fermented with co-culture seemed to have a higher content of free amino groups in albumin (~1.3 M/g protein) and globulin (~0.03 M/g protein) fractions compared to the mono-culture samples (albumin: ~1 M/g protein and globulin: ~0.02 M/g protein). Higher content of free amino groups in fermented samples could be explained by the proteolytic activity of the bacterial strains, leading to the release of peptides and amino acids during fermentation. A strong proteolytic activity was reported in some previous studies for *L. plantarum*. Rui et al. (79) reported the high proteolytic activity of *L. plantarum* in the fermentation of soy protein, causing an increase in the content of peptides. Oyedoh et al. (80) also observed high proteolysis and high concentration of peptides when cowpea was fermented with *L. plantarum*. *S. thermophilus* is known for its high proteolytic activity in milk (49). Moreover, there are some pieces of evidence of its proteolytic activity in legumes. For instance, Hati et al. (81) reported a maximum proteolysis and peptide generation for *S. thermophilus* in the fermentation of both bovine milk and soy milk. Boulay et al. (82) studied the role of cell envelope protease (CEP) in the growth of *S. thermophilus* in soy protein, and they observed a high proteolytic activity of *S. thermophiles*, resulting in the generation of more peptides and, consequently, a better growth of this strain. The increase in proteolytic activity of these LAB once in a co-culture has been reported previously. For instance, Madjirebaye et al. (83) observed an increase in the content of small peptides for the co-culture of *S. thermophilus* and *L. plantarum* compared to their mono-culture in the fermentation of soy milk. Li et al. (84) also reported a higher proteolytic activity of the co-culture of *S. thermophilus* and *L. plantarum* compared to the mono-culture of *S. thermophilus* or its co-culture with *B. animalis*, which led to the production of more free amino groups in fermented milk. Although the proteolytic activity and acid production capacity of the bacteria are known to be strain-dependent (85), there was no difference between the samples fermented with ST and LP. Despite the initial differences in the protein profile of the pea cultivars, there was no significant difference in their free amino group content. In albumin fraction, AS fermented with ST and LP had a slightly higher content of free amino groups content compared to the two other cultivars, but differences did not reach to be significant.

Additionally, the content of free amino groups was ~40–60 times lower in globulin fraction compared to the albumin ones, as the released peptides during fermentation are more soluble and are mostly recovered in the latter fraction.

### 3.3. Protein composition

**Figure 7:**
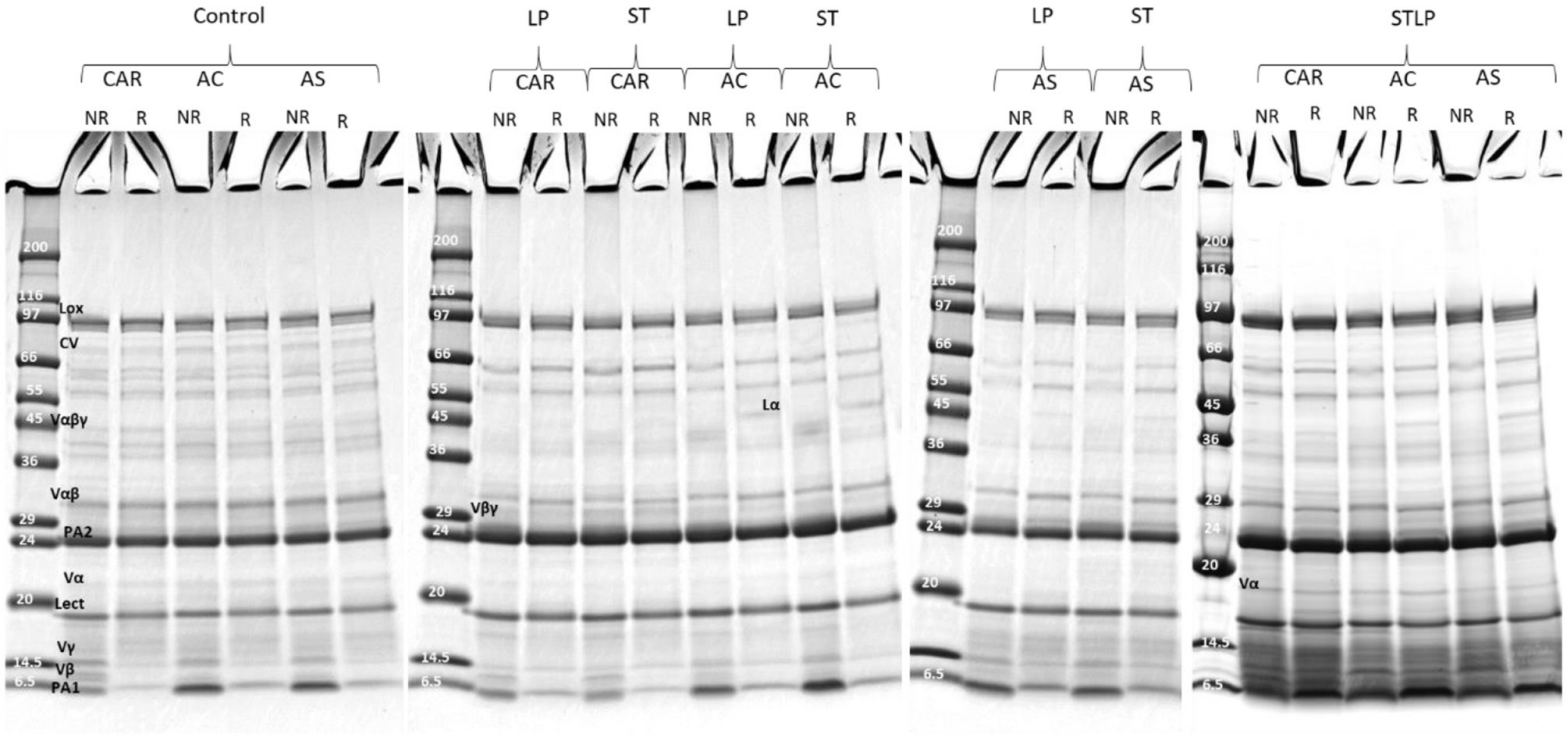
Electrophoresis profile of albumin fractions obtained without (control) or with added fermentation by *S. thermophilus* (ST), *L. plantarum* (LP), or their mixture (STLP). Comparison between different pea cultivars: CAR, AC, and AS in non-reducing (NR) and reducing (R) conditions.

SDS-PAGE was performed in non-reducing and reducing conditions to determine the effect of the extraction method on the protein composition of the recovered albumin and globulin fractions of different pea cultivars (CAR, AC, and AS). In non-reducing conditions (NR), the polypeptide profile of albumin (Figure 7) showed the presence of bands ranging from ~6 to ~99 kDa. In control, the bands corresponding to lipoxygenase (LOX, ~94 kDa) (86), convicilin (CV, ~71 kDa) (86), and vicilin monomer (Vα*βγ*, ~50 kDa) and the cleavage-resulting polypeptides (Vαβ, ~30–36 kDa; Vα, ~20 kDa; Vβ, ~13kDa; Vγ, ~12–16 kDa) (87, 88), lectine (Lect, ~17 kDa) (89), and the main 2S albumin subunits (PA2, ~26kDa; PA1, ~6kDa) (87, 90) were observed. While in fermented samples, CV and Vα*βγ* were absent. Other globulin contamination could be observed depending on LAB strains and cultivars. For instance, Vβγ (~25–30 kDa) was mainly observed in cultivars CAR and AC fermented with ST and LP. These two cultivars initially had a higher content of 7S globulin compared to cultivar AS. Different vicilin subunits (Vα, ~20 kDa; Vβ, ~13kDa; Vγ, ~12–16 kDa) were mainly observed for cultivars fermented with co-culture. The presence of legumin acidic subunit (Lα, ~40 kDa) (91), which was separated from legumin monomer (Lαβ ~60kDa) under reducing condition (R) (Figure 7), was observed for cultivars fermented with co-culture and cultivars AS and AC fermented with both ST or LP. The high intensity of bands smaller than 20 kDa in the electrophoretic profile of co-culture could indicate the presence of more peptides in this region. Production of small protein fractions and the disappearance of high Mw proteins was related to the proteolytic activity of LAB as revealed before by amino group quantification. The disappearance of high Mw protein in the electrophoretic profile after lactic acid fermentation has been previously shown in pea flour (92) and pea protein isolate (35). Boulay et al. (82) explained the proteolytic activity of ST in soy protein by the disappearance of high Mw protein and the presence of low Mw compounds in the polypeptide profile.

**Figure 8:**
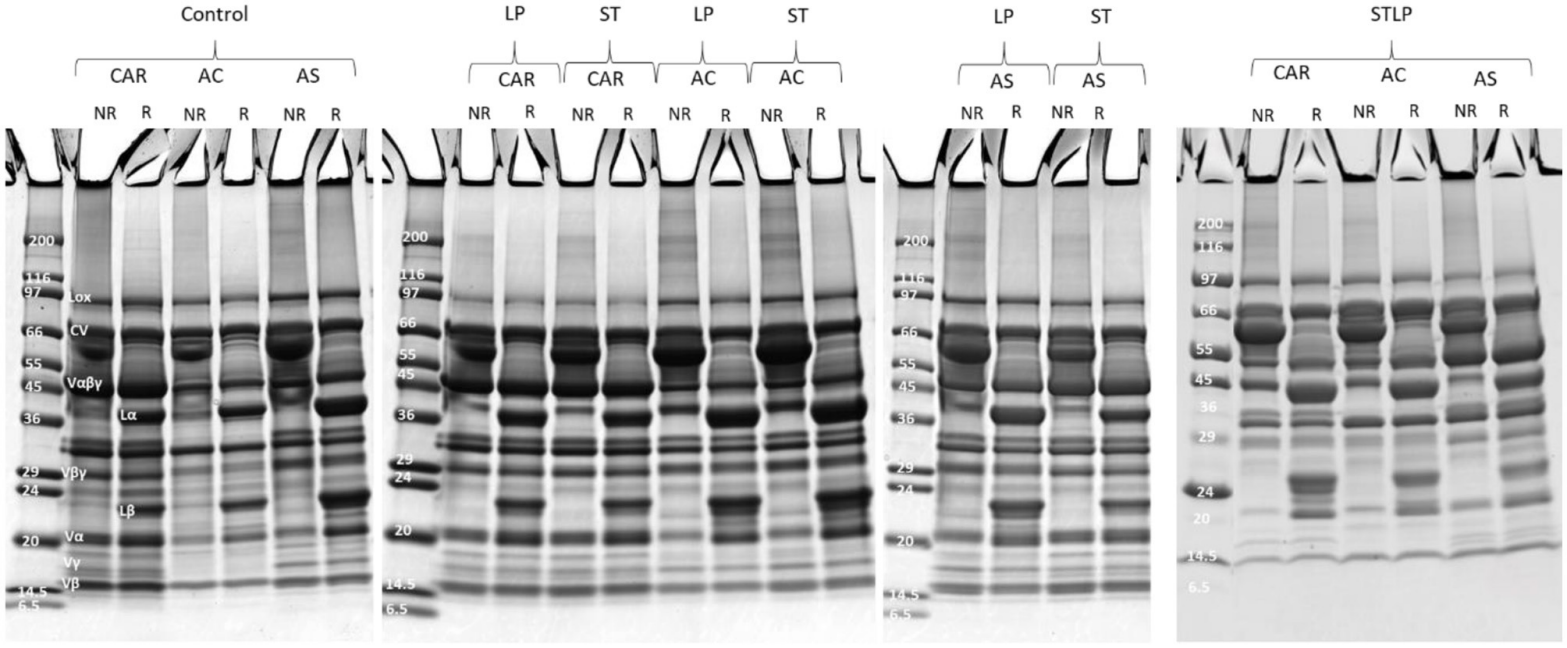
Electrophoresis profile of globulin fractions obtained without (control) or with added fermentation with *S. thermophilus* (ST), *L. plantarum* (LP), or their mixture (STLP). Comparison between different pea cultivars: CAR, AC, and AS in non-reducing (NR) and reducing (R) conditions.

The polypeptide profile of globulin fraction (Figure 8) in non-reducing conditions revealed the presence of bands ranging from ~10 to ~99 kDa. In globulin fraction, the 2S albumin subunits (PA2, ~26kDa; PA1, ~6kDa) (87, 90) were clearly absent for all the samples, while different groups of Vα*βγ* (~50 kDa) and derived subunits, Lαβ (~60 kDa) and its acidic (Lα, ~40 kDa) and basic (Lβ, ~20 kDa) subunits, CV, and LOX were observed for all the samples. As expected, the band corresponding to Lαβ disappeared under reducing conditions while Lα and Lβ bands enlarged significantly. The band corresponding to Vγ (~12–16 kDa) was absent in cultivar AC obtained without fermentation (control) and with co-culture, while this fraction was present for AC obtained by ST and LP. This could be related to the hydrolysis of vicilin monomer by ST and LP. The bands corresponding to vicilin subunits (<24 kDa) in the globulin fraction of co-culture seemed to be narrow, which might also result from the proteolysis of vicilin. At the same time, this could explain why different groups of vicilin subunits were observed in albumin fractions related to co-culture samples.

### 3.4. Size distribution of peptides

**Figure 9:**
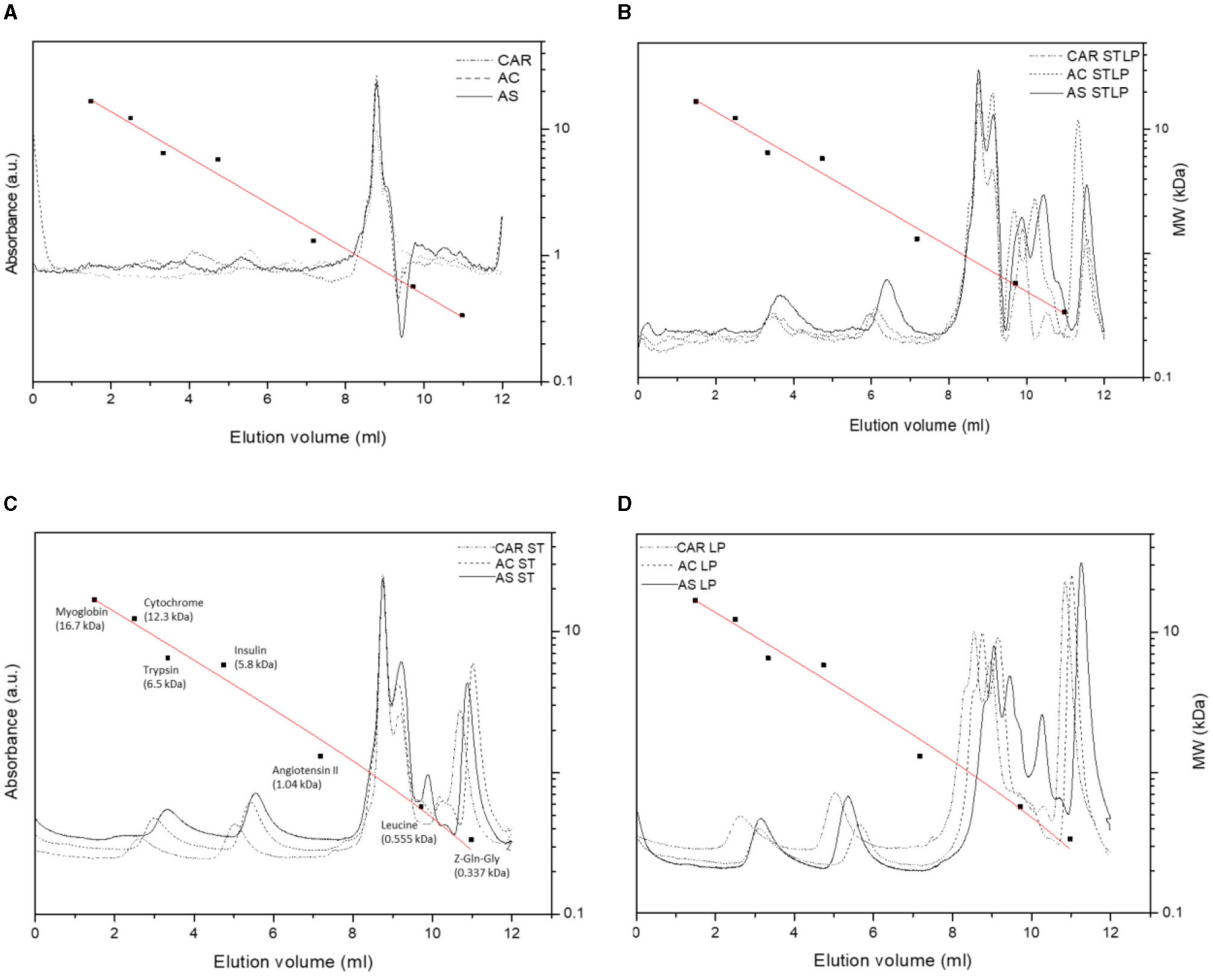
SEC-HPLC chromatograms of albumin fractions obtained without (control) **(A)** or with added fermentation by *S. thermophilus* (ST) **(C)**, *L. plantarum* (LP) **(D)**, or their mixture (STLP) **(B)**. Comparison between different pea cultivars: CAR, AC, and AS. The calibration curve was represented as a red line. Standards names with their corresponding Mw (■) were given in **(C)**. The calibration curve equation and the correlation coefficients were y = −0.18x + 1.503, R2 = 0.986.

SEC-HPLC was performed to study in detail the Mw distribution of peptides (<10 kDa) in the albumin fraction. Figure 9 showed the representative chromatograms for albumin fractions obtained without (control) and with fermentation with ST, LP, and STLP, by comparing the three different pea varieties. Seven different classes of Mw were distinguished in terms of their elution volume. The area of the peaks corresponding to each class was integrated to define the proportions corresponding to the different fractions, considering their sum equal to 100% (Table 3).

**Table 3:** Percentage of the integrated area of SEC-HPLC chromatograms at different elution volumes for albumin fractions obtained without (control) or with added fermentation by *S. thermophilus* (ST), *L. plantarum* (LP), or their mixture (STLP).

**Elution volume (mL)**	**~4.0**	**~6.0**	**~8.0**	**~9.0**	**~10.0**	**~10.5**	**~11.5**
**Mw (kDa)**	~**9.0–5.5**	~**1.8–4**	~**0.8–0.7**	~**0.7–0.6**	~**0.5–0.4**	~**0.4–0.3**	~**0.3–0.2**
CAR control	Trace	0.6 ± 0.0a^a^	0.8 ± 0.0a	ND	ND	ND	ND
AC control	0.5 ± 0.0a	0.6 ± 0.0a	0.8 ± 0.0a	ND	ND	ND	ND
AS control	0.5 ± 0.0a	0.4 ± 0.0a	0.9 ± 0.0a	ND	ND	ND	ND
CAR ST	9.0 ± 0.2c	3.3 ± 0.0b	12.5 ± 0.6d	11.3 ± 0.3b	ND	9.8 ± 0.5a	9.8 ± 0.9b
AC ST	15.7 ± 0.5f	11.6 ± 0.1d	11.8 ± 0.7d	7.7 ± 0.5a	ND	11.4 ± 0.6b	8.8 ± 0.8ab
AS ST	13.2 ± 0.4de	8.0 ± 0.1c	11.5 ± 0.7d	11.0 ± 0.4b	3 ± 0.2a	Trace	10.3 ± 0.7bc
CAR LP	13.5 ± 0.6e	13.0 ± 0.1e	9.4 ± 0.4c	6.7 ± 0.3a	ND	Trace	9.8 ± 0.8b
AC LP	8.2 ± 0.1c	3.5 ± 0.0b	7.2 ± 0.6b	6.5 ± 0.5a	ND	Trace	7.9 ± 0.7a
AS LP	12.3 ± 0.5d	7.2 ± 0.1c	8.9 ± 0.4bc	6.3 ± 0.4a	3 ± 0.3a	Trace	11.7 ± 0.7cd
CAR STLP	1.8 ± 0.0b	14.7 ± 0.2f	11.9 ± 0.6d	16.7 ± 0.9d	31.6 ± 0.48b	23.2 ± 0.9c	13.2 ± 0.8de
AC STLP	1.5 ± 0.0ab	13.5 ± 0.3e	11.6 ± 0.5d	18.9 ± 0.7e	31.3 ± 0.5b	23.6 ± 0.6c	14.8 ± 0.7e
AS STLP	1.3 ± 0.0ab	20.9 ± 0.5g	12.4 ± 0.6d	14.4 ± 0.6c	30.8 ± 0.5b	31.5 ± 0.9d	14.8 ± 0.6e

Comparison between different pea cultivars: CAR, AC, and AS.

ND, not detected by SEC-HPLC.

a Proportion of integrated area was presented as average ± standard deviation, n (n = 3). Different letters in the same column represent significant differences among different samples (Tukey's *post-hoc* test, P < 0.05).

The elution profiles of pea albumin fractions corresponding to control samples (Figure 9A) showed three major peaks (from ~4 to ~10 mL). Two minor peaks at ~,4 and 6 mL elution volume were related to the highest Mw protein components (>2 kDa) of the samples. These two peaks could be assigned to the PA1 protein, which was also observed in the electrophoresis pattern of all the samples at Mw at ~4–6 kDa. These calculated values matched with the ones from the literature (93, 94). The percentage of the integrated area of these two peaks was significantly lower for control (~0.5%) compared to the fermented samples (~13%) (Table 3), which could indicate the occurrence of new polypeptides resulting from LAB proteolytic activity. The third peak was the sharp one, which was observed for all the samples at ~8 mL elution volume representing peptides with Mw of ~0.8 kDa, representing ~0.8% of the integrated area for the control samples. This peak had a higher integrated area for the samples fermented with STLP and ST (~12%) compared to LP (~7.2–9.4%). The four other peaks at higher elution volumes (~9, ~10, ~10.5, and ~11.5 mL) representing lower Mw peptides and amino acids (<0.8 kDa) were only present for fermented samples. The proportion of these peaks was significantly different between the strains and cultivars (Table 3). Fermentation with co-culture led to a higher proportion of these peaks compared to the mono-cultures. A peak at ~10 mL elution volume was only observed for samples fermented with STLP (~31%) and cultivar AS fermented with ST and LP (~3%). In the last case (i.e., for AS), the proportion of this peak seemed to be higher in co-culture compared to ST and LP mono-cultures. The next two peaks at around 10.5 (STLP: ~23–30%, ST: ~9–11%, LP: trace) and 11.5 (STLP: ~13–14%, ST: ~9–10%, LP: ~7–11%) mL elution volume were detected for all the samples. These peaks had higher integrated areas for the samples fermented with STLP compared to mono-cultures. To resume, fermentation, especially with the co-culture, released small oligopeptides/peptides (with Mw ranging from 0.8 to 0.2 kDa) in a higher proportion compared to the controls. These results are in agreement with those obtained before in the SDS-PAGE pattern, and NPN and free amino group content reveal a proteolytic effect. The majority of these peptides were in the range of 0.8 to 0.2 kDa.

Regarding cultivars, the most significant differences were observed at elution volumes ~4 and ~6 mL which belonged to the polypeptides higher than 2 kDa. At elution volume ~4, the highest value was related to AC fermented with ST, followed by CAR fermented with LP. While cultivar AS seemed to have a richer profile in lower Mw peptides than the other two cultivars. Higher production of small peptides and amino acids in AS cultivars was coherent with the results obtained by NPN and free amino group content data.

### 3.5. Trypsin inhibitor activity

**Figure 10:**
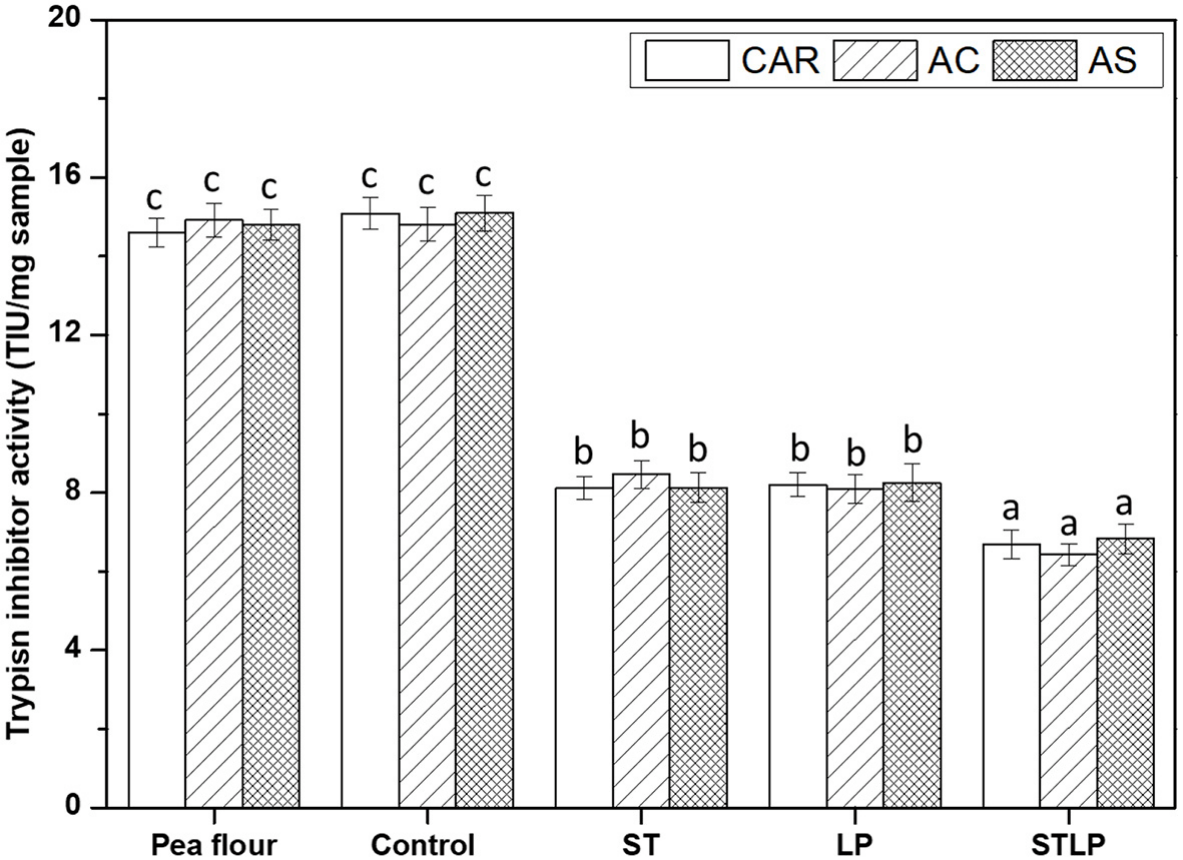
Trypsin inhibitor activity (trypsin inhibitor unit/mg sample) of albumin fractions obtained without (control) or with added fermentation by *S. thermophilus* (ST), *L. plantarum* (LP), or their mixture (STLP). Comparison between different pea cultivars: CAR, AC, and AS. Different letters represent significant differences among different samples (Tukey's *post-hoc* test, *P* < 0.05).

Trypsin inhibitor activity (TIA) was measured for pea flours of three cultivars (CAR, AC, and AS) and the corresponding albumin fractions obtained without (control) or with fermentation with ST, LP, or their co-culture (STLP) (Figure 10). The amount of TIA in pea flours was around ~14.8 TIU/mg sample with no significant differences between the cultivars. In general, pea is known for having a lower amount of trypsin inhibitor compared to other legumes (8). However, the amount of TIA can be significant in some cultivars and it can vary from 1 to 15 TIU/mg sample depending on the cultivars (95–97). The analysis also showed that the value of TIA in control (~15 TIU/mg sample) of albumin fraction samples did not change significantly from pea flours. This result confirmed the fact that trypsin inhibitor was recovered in the albumin fraction during extraction (98, 99). However, the TIA decreased to around ~8 TIU/mg sample for the fermented albumin fraction samples with again no significant difference within pea varieties. This result might be related to the proteolytic activity of LAB that could affect the native protein structure of trypsin inhibitors. The decreased level of TIA after fermentation with LAB in pea has been reported previously. For instance, Ma et al. (100) reported a reduction of ~50% in TIA of pea seeds fermented with a mixed LAB culture (containing *S. thermophilus, L. bulgaricus*, and *L. acidophilus*). Cabuk et al. (27) observed that the content of TIA in pea protein isolate dropped around 18% to 50% (as the fermentation time increased) after fermentation with *L. plantarum*. Byanju et al. (101) reported the fermentation of green pea with *L. plantarum* reduced TIA by ~47%. In the present study, no significant difference was observed between the mono-culture samples. However, the value of TIA was lower for the samples fermented with co-culture (~6.8 TIU/mg sample) compared to the mono-culture samples, pointing out again higher proteolytic activity of STLP compared to ST or LP alone; as it was previously observed in soy milk fermentation with co-culture of *S. thermophilus* and *L. plantarum* by Madjirebaye et al. (83).

### 3.6. Antioxidant activity

**Figure 11:**
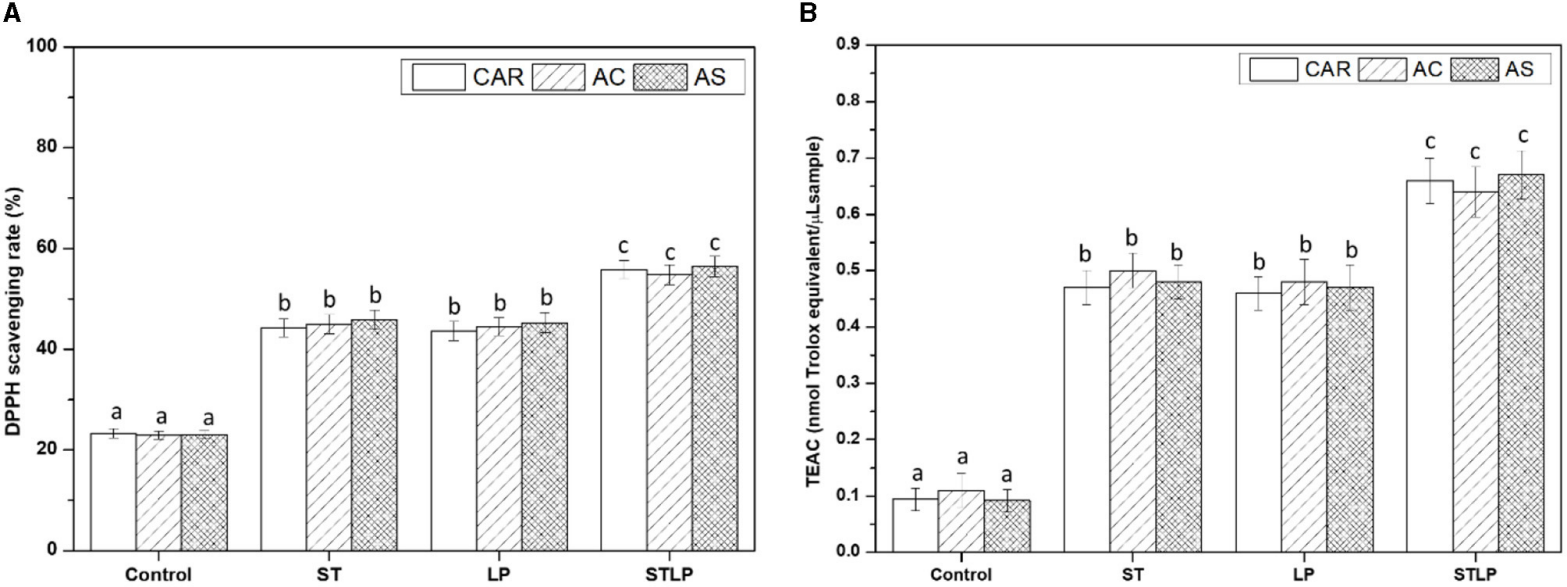
DPPH radical scavenging capacity **(A)** and Trolox equivalent antioxidant capacity (TEAC) **(B)** of albumin fractions obtained without (control) or with added fermentation by *S. thermophilus* (ST), *L. plantarum* (LP), or their mixture (STLP). Comparison between different pea cultivars: CAR, AC, and AS. Different letters represent significant differences among different samples (Tukey's *post-hoc* test, *P* < 0.05).

The antioxidant activity of albumin samples obtained without (control) and with fermentation with ST, LP, and their co-culture (STLP) was evaluated to determine whether the evidenced production of small peptides could induce enhanced antioxidant activity. As shown in Figure 11, DPPH scavenging activity (Figure 11A) and TEAC (Figure 11B) in fermented samples was ~2–3 times higher than control. This indicated a correlation between the two methods used for measuring antioxidant activity. The value of DPPH scavenging was ~23% in control, while this value was ~45% for mono-cultures and ~56% for co-cultures. TEAC was ~0.1 (nmol Trolox equivalent/μL sample) for control. In fermented samples, this value was ~0.5 (nmol Trolox equivalent/μL sample) for mono-cultures and ~0.65 (nmol Trolox equivalent/μL sample) for co-cultures. The improved antioxidant activity for fermented samples could be associated with the production of peptides during fermentation. Release of small peptides with antioxidant activity from legumes has been reported several times. For instance, Leksono et al. (102) reported an increase in the antioxidant activity of black soybean milk fermented with *S. thermophilus* and *L. plantarum* mono-cultures. They also observed a similar pattern in increasing the antioxidant activity between the strains. Sáez et al. (103) observed that chickpea fermented with *L. plantarum* had a 40% increase in antioxidant activity compared to control. Torino et al. (104) also reported an increase in the antioxidant capacity of lentil fermented with LP. Naprasrt et al. (105) observed that the antioxidant activity of fermented red bean milk was two times higher than unfermented red bean milk, and the highest value among different LAB was related to *L. plantarum*. Contrary to the results of the present study, Lee et al. (106) reported that, in the fermentation of black soymilk with *S. thermophilus* and its co-culture with *L. plantarum*, the highest antioxidant activity belonged to mono-culture compared to co-culture.

### 3.7. Phytic acid

**Figure 12:**
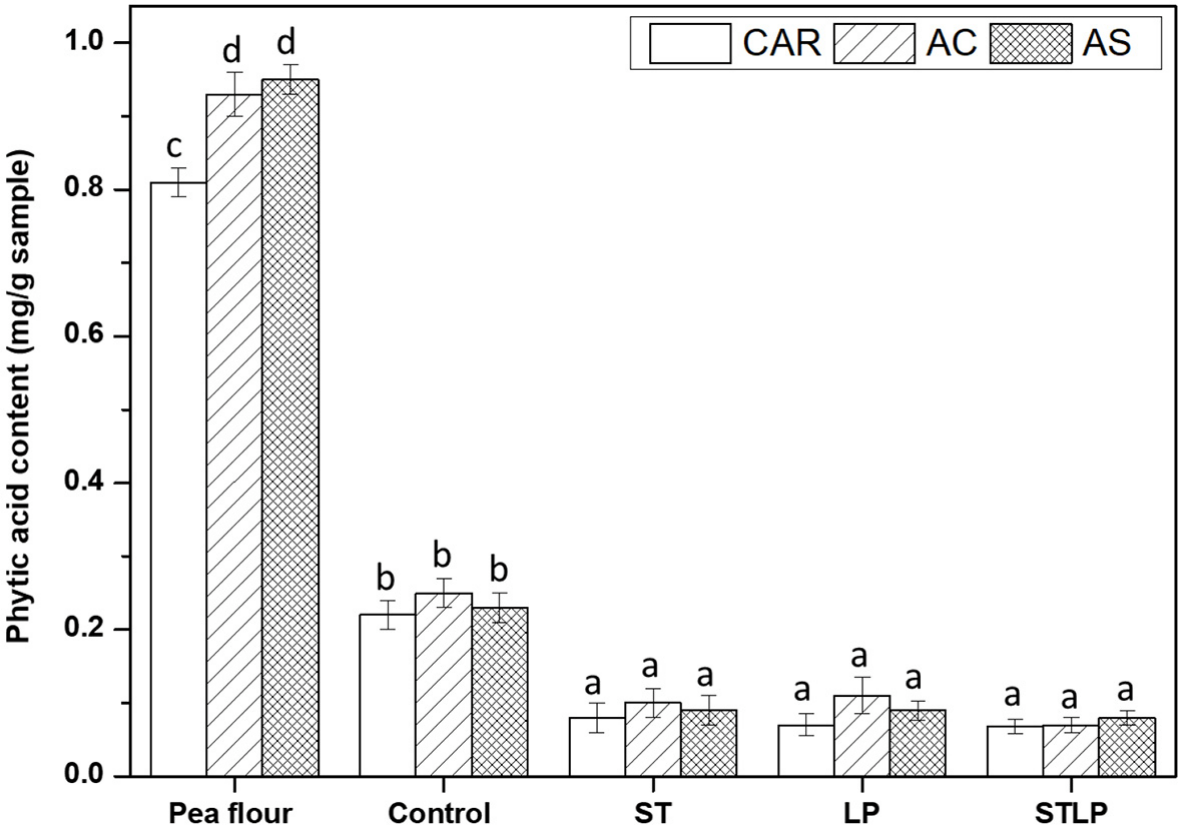
The phytic acid content of albumin fractions obtained without (control) or with added fermentation by *S. thermophilus* (ST), *L. plantarum* (LP), or their mixture (STLP). Comparison between different pea cultivars: CAR, AC, and AS. Different letters represent significant differences among different samples (Tukey's *post-hoc* test, *P* < 0.05).

Phytic acid content was measured for pea flour of the three cultivars (CAR, AC, and AS), and the corresponding albumin fractions were obtained without (control) or with fermentation with ST, LP, or their mixture (STLP) (Figure 12). The content of phytic acid in pea cultivars AS and AC was ~0.9 mg/g of flour sample on a dry basis, while this value was significantly lower in CAR (~0.8 mg/g flour). In addition, there was a significant reduction in the amount of phytic acid for albumin fractions in control (~0.2 mg/g freeze-dried albumin sample) compared to flour. It could be possible that, during the extraction of pea protein, phytic acid initially present in protein bodies was not totally solubilized or that insoluble complexes were formed with other compounds such as globulins and divalent cations (11). The reduction of phytic acid in albumin fractions can also be explained by the endogenous phytase activity of legumes, as phytase is primarily located in the protein bodies (107). The content of phytic acid in fermented (~0.06–0.09 mg/g freeze-dried albumin sample) samples was 2–3 times lower than control. The degradation of phytic acid in fermented samples could be mainly related to the microbial phytase activity of both ST and LP (108). LP and ST are both able to produce phytase. Sumengen et al. (109) reported high intra- and extra-cellular phytase activity for *L. plantarum*. A high phytase activity was also reported for *S. thermophilus* by Priyodip and Balaji (110, 111). However, in the present study, no significant differences were observed between the strains, which could suggest the similar activity of phytase among ST and LP. Several articles have proven the phytic acid reduction in other fermented legume samples by these bacteria species. For instance, Xing et al. (112) showed the degradation of phytic acid in chickpea protein concentrate after fermentation with different LAB species. Fritsch et al. (30) also observed a reduction of phytic acid content by more than half in lupin protein isolate fermented with *L. plantarum*. Rui et al. (113) reported a reduction of 50% in the content of phytic acid (~3 mg/g sample) for soy seeds fermented with *L. plantarum*. Mefleh et al. (114) reported ~70% reduction in phytic acid content for chickpea protein isolate fermented with *S. thermophilus* mono-culture and its co-culture with *L. plantarum*.

## 4. Conclusion

The results from the study supported the hypothesis that AEIEP extraction assisted by LAB fermentation improves the nutritional quality of albumin fraction extracted from peas compared to the traditional AEIEP method without impairing the protein profile of the globulin fraction. Using ST, LP, and their co-culture allowed modifications in protein composition by increasing nitrogen and peptide contents, increasing antioxidant activity, and reducing antinutritional compounds such as trypsin inhibitors, α-galactosides, and phytic acid. These effects were mainly associated with the enzymatic activity of the selected LAB strains during fermentation. However, these changes depended on the microorganism used. In particular, the co-culture showed the highest level of proteolysis and the highest production of small peptides, which was related to the synergetic effect of the bacteria. Moreover, the main properties studied in this report did not seem to be pea genotype-dependent. These results indicate the possibility of tailoring properties of the still underutilized albumin fraction depending on its application. Despite the nitrogen enrichment of this fraction, the protein content remains low (~25–30%) to be considered as a protein ingredient, indicating the necessity for additional purification. While the primary focus of this study centered on the nutritional aspects of the albumin fraction, it would be intriguing to explore the presence of these antinutritional compounds in the globulin fraction as well, which is the most used fraction. Additionally, evaluating the physicochemical and functional properties of both fractions is essential to comprehensively assess the impact of fermentation. Furthermore, considering fermentation as a means to enhance the sensory attributes of these pea protein fractions could be advantageous for their increased utilization in plant-based food products.

## Data availability statement

The original contributions presented in the study are included in the article/supplementary material, further inquiries can be directed to the corresponding author.

## Author contributions

ME: Investigation, Methodology, Validation, Visualization, Writing—original draft. SM: Investigation, Methodology, Validation, Visualization, Writing—review & editing. BO: Conceptualization, Methodology, Supervision, Writing—review & editing. RS: Conceptualization, Methodology, Project administration, Supervision, Writing—review & editing.
